# Variation in Service Life on RC Structure According to Concrete Binder Type

**DOI:** 10.3390/ma13235430

**Published:** 2020-11-28

**Authors:** JangHyun Park, JinHo Park, MyeongGyu Jung

**Affiliations:** Korea Institute of Future Convergence Technology, Hankyong National University, 327 Jungang-ro, Anseong 17579, Korea; parkjh@hknu.ac.kr (J.P.); parkjh9422@hknu.ac.kr (J.P.)

**Keywords:** reinforced concrete, additive, corrosion, chloride ion diffusion coefficient, critical chloride content, service life

## Abstract

When an additive is used to replace a certain amount of cement, a concrete pore structure becomes dense. Thus, it results in inhibiting the penetration of chlorine ions and suppressing corrosion of reinforcing bars. However, the pH level of the concrete decreases, and it deteriorates the performance of the passive film formed on the surface of the rebars embedded in the concrete. Therefore, in this study, the service lives of reinforced concrete containing different types of concrete binders were predicted and compared. The chloride ion diffusion coefficients of concretes with various binders were measured, and the critical chloride content of the rebar was assessed by the real-time monitoring on the corrosion initiation time of the rebar embedded in concrete. Moreover, Fick’s 2nd law was applied to predict when the chloride content at the 40 mm depth of cover reached the critical chloride content based on the chloride ion diffusion coefficient. It was observed that the service life of S6 (OPC 40% + GGBFS 60%) was the highest, followed by TBC (OPC 40% + GGBFS 40% + FA 20%), S3 (OPC 70% + GGBFS 30%), and OPC (OPC 100%).

## 1. Introduction

With the durability of concrete emerging as a social issue, the need to develop concrete with high durability has been coming to the fore. Of particular concern are concrete structures in areas affected by the marine environment are subject to immense maintenance costs due to the corrosion of rebars caused by the penetration of chloride ions [1,2].

In recent years, the demand to construct concrete structures around port facilities has increased due to the increase of goods transported by sea, so it is becoming important to build concrete structures with excellent resistance to marine environments both in terms of structural stability and in terms of economics [3,4,5].

Methods using such admixtures as ground granulated blast furnace slag (GGBFS) and fly ash have been proposed to improve the durability of concrete exposed to chloride attack [6,7,8,9,10,11,12,13,14,15,16,17,18,19,20,21,22,23,24,25]. In particular, replacing a portion of cement with GGBFS can suppress the penetration of chloride ions by densifying the concrete pore structure due to latent hydraulic reaction [13,14,15,16,17,18,19,20,21,22,23,24,25]. It is also reported that the use of admixtures can suppress the corrosion of rebars by reducing the number of water-soluble chloride ions in the pore solution by fixing the penetrating salt on the cement matrix [6,7,8,9,10,11,12].

However, studies have shown that replacing the cement used in concrete with admixtures such as GGBFS and fly ash can reduce the critical chloride content of rebars embedded in the concrete [26,27,28,29,30,31,32,33,34]. It is critically important to correctly determine the critical chloride content of rebars in order to ensure the durability of reinforced concrete structures, as an incorrect estimation of critical chloride contents can cause errors in the design for durability against chloride attack [35,36,37,38,39]. Therefore, it is necessary to thoroughly evaluate the durability performance of concrete structures according to the types and replacement ratios of admixtures. Moreover, it is also essential to evaluate the chloride ion diffusion coefficient and the critical chloride content of rebars in concrete containing admixtures to assess and predict the durability of concrete [40,41,42,43,44]. A lot of research has been done on the critical chloride content of rebars embedded in the concrete or on the chloride ion diffusion coefficient of concrete depending on the types of concrete binders [45,46,47,48,49]. In addition, research has been conducted to predict the service life of the concrete by using data from previous publications [50,51,52,53]. However, few studies have predicted the service life of concrete by measuring the amount of critical chloride content on the reinforced concrete and the chloride ion diffusion coefficient of the same concrete specimen.

In this study, the variation of the service life of reinforced concrete was evaluated according to the different types and replacement ratios of additives. The chloride ion diffusion coefficient of concrete was measured based on NT BUILD 492. In addition, the corrosion initiation time and the critical chloride content of the rebar embedded in reinforced concrete were assessed by using an embedded reference electrode with MnO_2_. The penetration rate of chloride ions was calculated using Fick’s 2nd law, and the time when the chloride content at the 40 mm depth of cover reached critical chloride content was considered as the point at which the service life of concrete terminated.

## 2. Materials and Specimens

### 2.1. Materials

Ordinary Portland Cement (Type 1) of ASTM C150/C150M [54] with a density of 3.15 g/cm^3^ (S company, Seoul, Korea) was used. And Grade-80 Ground Granulated Blast Furnace Slag (GGBFS) of ASTM C989/C989M [55] with a Blaine fineness of 4000 cm^2^/g (Ssangyong company, Seoul, Korea) was used. Class-N Fly-Ash (FA) of ASTM C 618 [56] with a Blaine fineness of 3000 cm^2^/g was also used. Table 1 shows the chemical compositions of cement and additives.

To reduce the effect of coarse aggregates between the rebar and cover of concrete, the maximum size of coarse aggregates was limited to 13 mm and the fine aggregates were prepared by mixing crushed sand and washed sea sand. In Korea, it is common to use a combination of crushed aggregates and washed sea sand (decontaminated chloride) owing to the depletion of river sand [57,58]. The superplasticizer (S.P.) and air-entering (A.E.) and high-range water-reducing agent were used together to ensure the fluidity and air contents of concrete. Also, to control the effects of chloride ion in the chemical admixtures (Ssangyong company, Seoul, Korea) was used, which did not contain chloride.

### 2.2. Concrete Mix Proportion

For this study, four types of concrete specimens were prepared according to the GGBFS and FA replacement ratio: OPC 100% (OPC), GGBFS 30% + OPC 70% (S3), GGBFS 60% + OPC 40% (S6) and GGBFS 30% + FA 30% + OPC 40% (TBC).

As for the experimental level, normal GGBFS cement (replacement ratio 30%), high durability GGBFS cement (replacement ratio 60%), and three-binder combination cement (replacement ratio 60%) were compared with 100% OPC. Table 2 shows the concrete mix proportions according to the experiment levels.

### 2.3. Concrete Specimens

#### 2.3.1. Concrete Specimen for Measurement of Chloride Ion Diffusion Coefficient of Concrete

**Figure 1:**
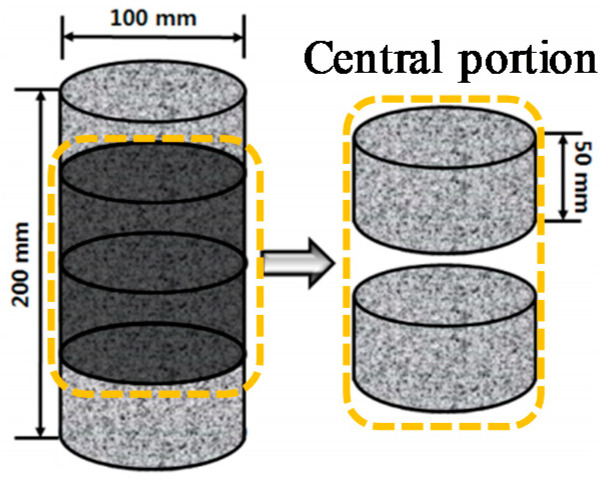
Schematic diagram of specimen for a chloride penetration test.

After the concrete was mixed for specimen fabrication, it was poured into a cylinder mold (Ø100 mm × 200 mm) and sealed. The specimen was demolded 24 h later and cured for 28 days. After curing 28 days, the specimen was cut into samples 50 mm and the 2 samples from the middle of the specimen were used. Figure 1 shows the schematic diagram of the specimens for measurement for chloride ion diffusion coefficient [59].

#### 2.3.2. Specimen for Measuring the Critical Chloride Contents of Rebars Embedded in Concrete

**Figure 2:**
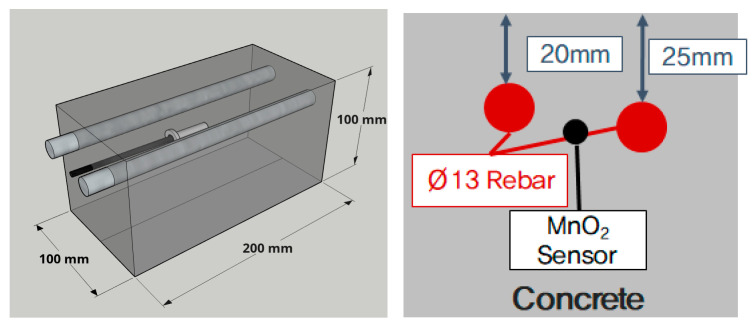
Schematic diagram of specimen for monitoring corrosion potential test.

Reinforced concrete specimens were fabricated to evaluate the critical chloride contents of rebars according to the concrete mixture. The reinforced concrete specimens were prepared by placing rebars in a rectangular mold (size: 100 mm × 100 mm × 200 mm) using acrylic, arranging the MnO_2_ sensor, and finally placing concrete. Figure 2 shows a schematic and cross-sectional diagram of the fabricated reinforced concrete specimens.

SD400 rebars (Ø13 mm) were used to fabricate the specimens. The foreign substances and corrosion products on the surface of the rebars resulting from long-term exposure to air were removed by using grit-blast and sandpaper. The MnO_2_ sensor was placed close between two rebars and embedded in concrete along with the rebars to allow more accurate measurement with fewer errors.

#### 2.3.3. Curing Method

After mixing the concrete, it was poured into the mold and demolded after 24 h. Then, the concrete specimens were cured by air-dry curing for 28 days. The air-dry curing conditions were a temperature of 20 °C ± 2 °C and relative humidity of 60% ± 5%. In order to simulate the curing conditions of the concrete poured on the site, air-dry curing was performed, not under-water curing.

## 3. Experiment

### 3.1. Compressive Strength Test

The concrete compressive strength was evaluated according to ASTM C 39 [60] for curing at 3 days, 7 days, and 28 days. Concrete specimens of Ø100 mm × 200 mm were prepared for each experimental level for the compressive strength test. The concrete compressive strengths of three specimens were measured and their average value was used.

### 3.2. Measurement of Resistance to Chloride Ion Coefficient of Concrete

**Figure 3:**
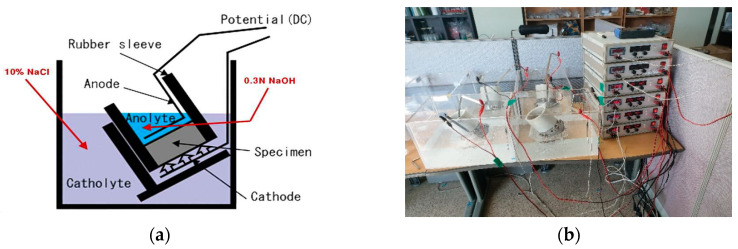
(**a**) Schematic diagram of NT BUILD 492 test, (**b**) Photograph image of the experimental setup.

The most exact method of measuring the resistance to chloride ingress of concrete is to perform an exposure test. However, exposure tests required a very long time. Therefore, it is common to evaluate the chloride ion diffusion coefficient and resistance to chloride ingress of concrete using the electrochemical acceleration method. Typically, NT BUILD 492 and ASTM C 1202 are used to evaluate chloride ingress of concrete. In this study, among the resistance to chloride ingress evaluation methods, the chloride ion diffusion coefficient was obtained from non-steady-state migration experiments conducted according to NT BUILD 492 [59], a Nordic standard frequently used as a quantitative evaluation method. Figure 3 shows the schematic diagram of the NT BUILD 492 test cells configured for this experiment and a photograph image of the installed experimental setup.

NT BUILD 492 is a non-steady-state electric migration experiment to determine the chloride ion diffusion coefficient of the repair material composed of concrete, mortar, and cement. Concrete specimens (size: Ø100 mm × 50 mm) were prepared, Ca(OH)_2_ saturated solution was filled in a desiccator and the concrete specimen was immersed in the saturated solution. The inside of the desiccator was maintained in a vacuum state by using a vacuum pump, and the pores of the concrete were filled with Ca(OH)_2_ saturated solution. After pretreatment, chloride ion diffusion cells were prepared as shown in Figure 3. The anolyte was filled with 0.3 M NaOH and the catholyte was filled with 10% NaOH solution. Then, we measured the initial current value (*l*_30V_). Next, according to the initial values in Table 3, the current range was found, and the actual applied voltage was adjusted. The resistance to chloride ion penetration was tested using the potential difference by selecting an appropriate time according to the current range.

After the test, the concrete specimen was split vertically into two pieces. When 0.1 N AgNO_3_ solution was sprayed on the split section, discolored parts appeared on the specimen depending on the penetration depth of the chloride ions. The chloride ion diffusion coefficient was determined using the average of seven measurements of the chloride penetration depth, in 10 mm intervals.

Equation (1) is used to estimate the chloride ion diffusion coefficient at the depth of chloride ion penetration [59].

(1) $$D_{nssm}\;=\;\frac{0.0239\left(273+T\right)L}{\left(U-2\right)t}\left(x_{d}-0.0238\sqrt{\frac{\left(273+T\right)Lx_{d}}{U-2}}\right)$$

where *D_nssm_*: non-steady-state migration coefficient (×10^−12^ m^2^/s), *U*: absolute value of the applied voltage (V), *T*: average of the initial and final temperatures in the anolyte solution (°C), *L*: thickness of the specimen (mm), *x*_d_: average value of the penetration depths (mm), *T*: test duration (h).

### 3.3. Potential Monitoring of Rebar Embedded in Concrete

**Figure 4:**
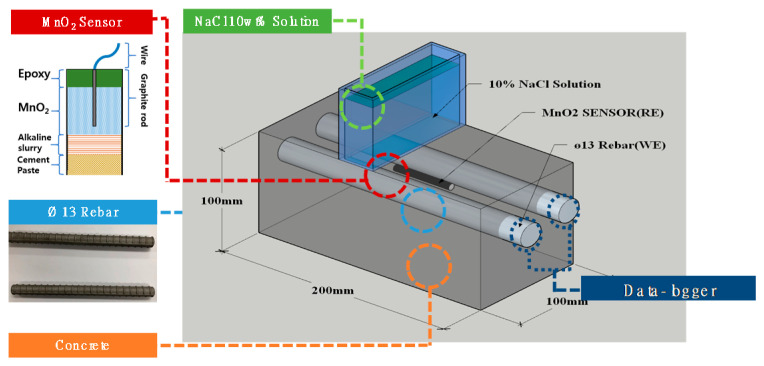
Schematic diagram of potential monitoring experiment according to NaCl supply time.

The potential (OCP) monitoring test of rebars was performed according to the NaCl supply time, and Figure 4 shows a schematic diagram of the experiment.

After connecting wires to the rebars of the specimen and the MnO_2_ sensor, an NaCl supply cell was installed on top of the specimen using acrylic, and the potential of the rebars was monitored in real-time using a Data-logger (Graphtec, GL-820, Yokohama, Japan) according to the supply time of 10 wt.% NaCl solution. The wires were soldered to the rebars to monitor changes in the potential of the rebars. The rebars were connected to the anode (+) of the data-logger and the MnO_2_ sensor to the cathode (−) to collect and store data by measuring the potential of the rebars to the MnO_2_ sensor every 10 min. While observing the potential of the rebars according to the 10 wt.% NaCl solution supply time, the rebars were evaluated as corroded if the potential of the rebars dropped below −515 mV, in which case, the test was ended by stopping potential measurement and the supply of NaCl solution [61]. Then, the specimen was split into two pieces to allow for a visual observation of the corrosion on the surface of the rebars [61,62].

Table 4 shows the criteria for determining rebar corrosion according to the potential of rebars by the type of reference electrode in ASTM C 876 used to determine rebar corrosion.

**Figure 5:**
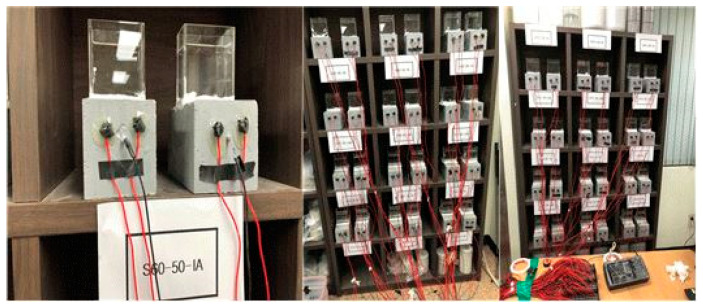
Photograph image of potential monitoring experiment.

Figure 5 shows an image of the potential monitoring experiment of the rebars embedded in concrete.

### 3.4. Evaluation of Critical Chloride Content of Rebar Embedded in Concrete

**Figure 6:**
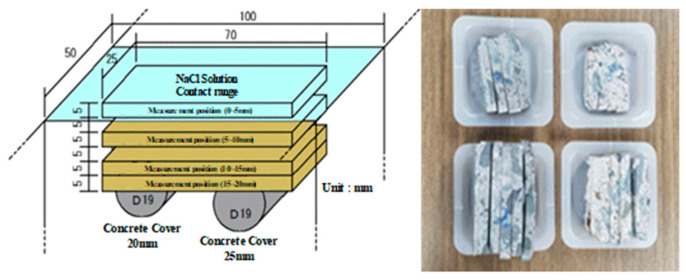
Test specimen collection method for measuring the amount of chloride in concrete.

Figure 6 shows a schematic diagram of the method for collecting concrete specimens to evaluate the chloride content by concrete cover depth and an image of the collected specimens.

**Figure 7:**
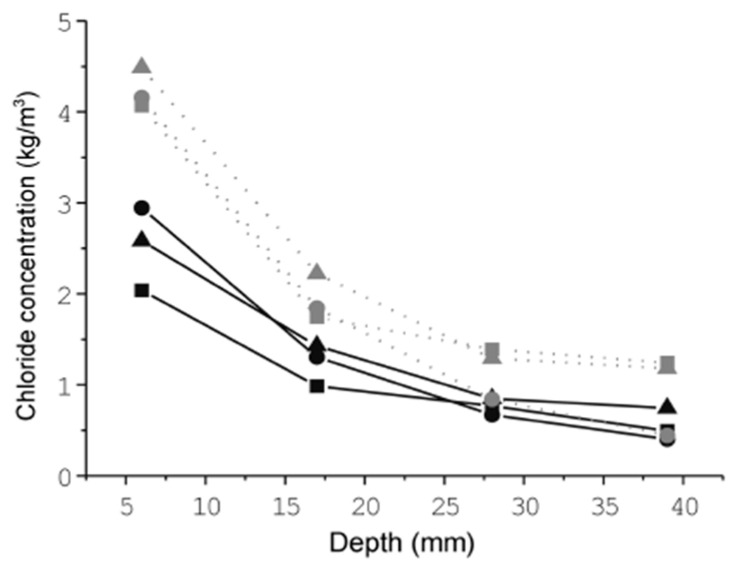
Chloride profiling of concrete according to concrete cover depth.

The supply of NaCl solution was stopped for the 20 mm concrete cover specimens in which rebar corrosion had started and the 25 mm concrete cover specimens in which rebar corrosion did not occur. The concrete specimens were collected after cutting the specimens at 5 mm intervals from the NaCl solution supply surface, and the chloride content was measured according to ASTM C 1218 [63] to profile the chloride content of concrete according to the cover depth as shown in Figure 7.

After measuring and profiling the chloride content according to the concrete cover depth, the chloride content between the 20 mm concrete cover depth of corroded rebars and the 25 mm concrete cover depth of non-corroded rebars was estimated as the critical chloride content of the rebars embedded in concrete.

Titration with silver nitrate was used to measure the chloride ion concentration in the concrete, and Equation (2) shows how to calculate the chloride content for the mass of concrete according to the appropriate amount of silver nitrate [63].

(2) $$Cl^{-}\;=\;\frac{3.545[\left(V_{1}-V_{2}\right)N]}{W}$$

where *V*_1_: Volume of 0.05 N silver nitrate solution used for titration in the specimen (mL, equivalence point), *V*_2_: Volume of 0.05 N silver nitrate solution used for blank titration (mL, equivalence point), *N*: The exact normal concentration of 0.05 N silver nitrate solution (N), *W*: The mass of the specimen (g).

After calculating the chloride content (%) for the mass of concrete, the chloride content (%) for the cement or binder was obtained by multiplying the chloride content (%) by 100/P. The *p*-value is the cement mass ratio (%) in mortar or concrete, and this study used the cement mass ratio.

### 3.5. Evaluation of Service Life of Reinforced Concrete Structure

Fick’s Second Law was used to evaluate the service life of the reinforced concrete structure, as shown in Equation (3) below [3,64].

(3) $$C\left(x,t\right)=C_{0}\left\{1-erf\left(\frac{x}{2\sqrt{D\times t}}\right)\right\}$$

where *C(x,t)*: Chloride ion concentration (kg/m^3^) at depth x (cm) and time y (year), *C*_0_: Chloride ion concentration on the surface (kg/m^3^), *D*: Chloride ion diffusion coefficient, *erf*: Error function

The cover depth of the rebars embedded in concrete was assumed to be 40 mm, and the service life of concrete was determined as the time until the chloride ion concentration on the surface reaches the critical chloride content. The test results in Section 3.2. were used as the chloride ion diffusion coefficient of concrete and the critical chloride content of the embedded rebars was calculated by using the critical chloride content (4% of binder) according to the Korean concrete standard specification and the test results in Section 3.3 to compare and evaluate the results.

## 4. Results and Discussion

### 4.1. Result of Concrete Compressive Strength

**Figure 8:**
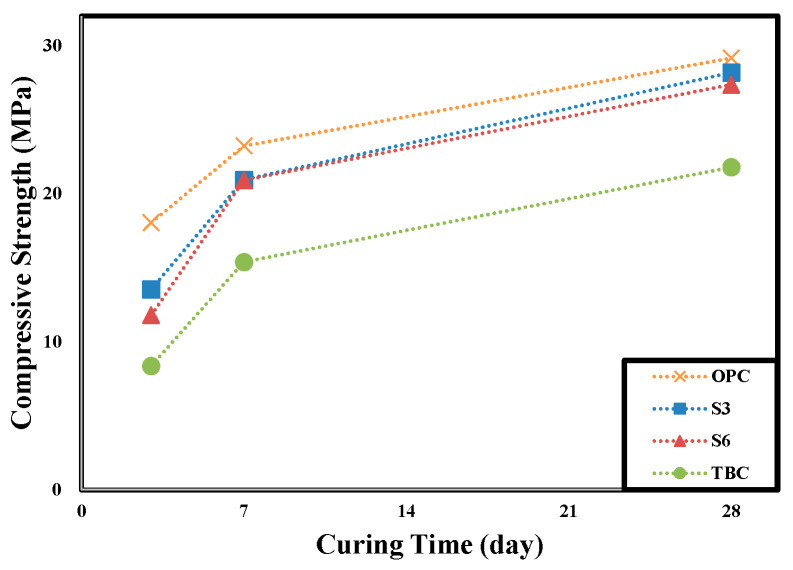
Variation of compressive strength on curing days by specimens.

Table 5 shows the results of measuring the concrete compressive strength of each specimen, and Figure 8 shows the concrete’s compressive strength according to the curing time.

The measured initial compressive strength (3d) of concrete can be ranked in the order of OPC > S3 > S6 > TBC, while the compressive strength after 28 days can be ranked in the order of OPC 29.2, S30 28.2, S60 27.4, and TBC 21.8 MPa, showing the same trend as the initial compressive strength. The compressive strength of concrete decreased as the admixture replacement rate increased, and with the same 60% replacement ratio, the GGBFS 60% specimen showed higher compressive strength than the TBC specimen. The development of concrete compressive strength was limited because of the insufficient hydration process under air-dry curing conditions and the absolute quantity of cement (40%), which reduced the production of Ca(OH)_2_ and limited the pozzolanic reaction of fly ash [7,12,13,15]. In the case of air-dry curing, the pozzolanic reaction of fly-ash due to insufficient curing water or the latent hydraulic properties of GGBFS are not properly exhibited. As a result, it is judged that the compressive strength of concrete was low. Further research is needed to verify the reduction of compressive strength development by performing a pore structure analysis and micro-analysis of concrete.

### 4.2. Result of Chloride Ion Diffusion Coefficient of Concrete

**Figure 9:**
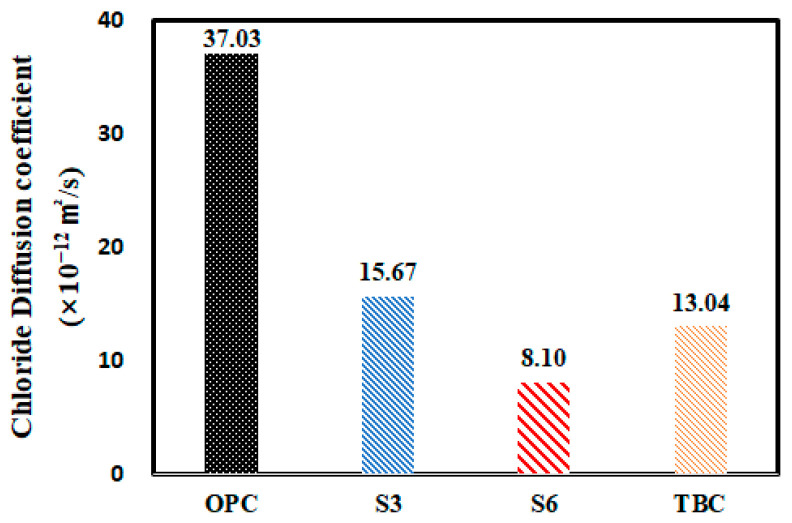
Chloride ion diffusion coefficient of concrete according to binder type.

Table 6 and Figure 9 show the results of measuring the chloride ion diffusion coefficient of concrete according to the experiment level. Although the chloride ion migration coefficient measured according to the non-steady-state electrophoresis method of NT BUILD 492 is different from the apparent diffusion coefficient, this method is commonly used because of the time required for measurement, and this study also used this value for the analysis.

According to the measurement results, the chloride ion diffusion coefficient of concrete can be ranked in the order of OPC > S3 > TBC > S6, and replacing OPC with GGBFS or FA was shown to be effective in reducing the number of ions penetrating the concrete. However, when replacing GGBFS 40% + FA 20%, the chloride ion diffusion coefficient was not significantly different from the GGBFS 30% specimen even though the replacement rate was 60%. This is because the latent hydraulic property of GGBFS and the pozzolanic reaction of FA did not occur sufficiently due to the decrease in the absolute quantity of cement and the hydration products under the high admixture replacement rate and air curing conditions, so the structure of the cement matrix was not completed properly [6,7,8,9,10,11,12,13,14,15,16,17,18,19,20]. As a result, the compressive strength of concrete decreased, along with the resistance to chloride penetration. For this reason, concrete pore structure analysis and micro-analysis should be performed in the future in addition to analyses according to the curing conditions and period.

### 4.3. Result of OCP Monitoring

**Figure 10:**
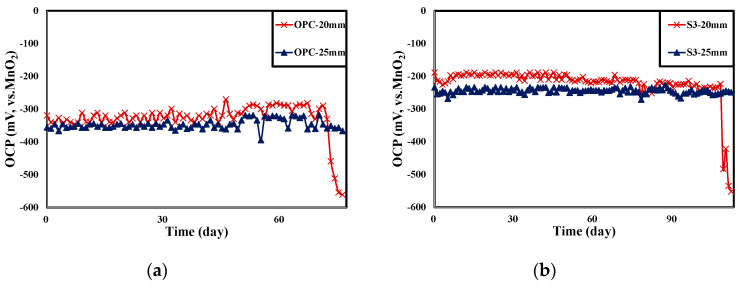

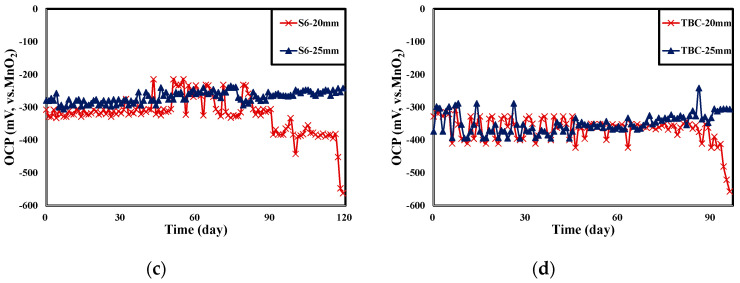
Result of OCP monitoring on NaCl supply time (**a**) OPC, (**b**) S3, (**c**) S6, (**d**) TBC.

Figure 10 shows the results of monitoring the potential of the rebars embedded in the concrete according to the NaCl solution supply time for each experiment level.

Table 7 shows the surface image of rebar after OCP monitoring experiment. The potential of the rebars with 20 mm concrete cover depth decreased to below −515 mV (vs. MnO_2_) as the NaCl solution supply time increased, and rebar corrosion occurred accordingly. On the other hand, the potential of the rebars with 25 mm concrete cover depth was stable and rebar corrosion did not occur. The time it took for the rebars with 20 mm concrete cover to corrode according to the NaCl solution supply time was 76 days for OPC, 96 days for TBC, 112 days for S3, and 119 days for S6, which has the same trend as was shown in the evaluation of the concrete chloride ion diffusion coefficient in Section 4.2. Through visually observing the surface of the rebars embedded in concrete, corrosion products were found in the rebars with 20 mm concrete cover depth in all of the specimens, while the surface of the rebars with 25 mm concrete cover depth remained intact, without corrosion. Although there was a difference in the degree of corrosion, determining the corrosion initiation point of the rebars by monitoring the potential of the rebars using a MnO_2_ sensor was considered to be valid and accurate. In addition, improving the precision of the MnO_2_ sensor should allow us to correct this difference in the future [62].

In particular, since the surface of the rebars with 25 mm concrete cover depth remained intact and without corrosion, the critical chloride content that induces rebar corrosion in concrete can be roughly estimated by measuring the chloride content in the 20–25 mm concrete cover.

### 4.4. Result of Critical Chloride Contents Measurement

The specimens in which the rebars started to corrode were cut at 5 mm intervals from the NaCl solution supply surface to collect the concrete samples and evaluate the amount of chloride content, and the results are shown in Table 8 below.

**Figure 11:**
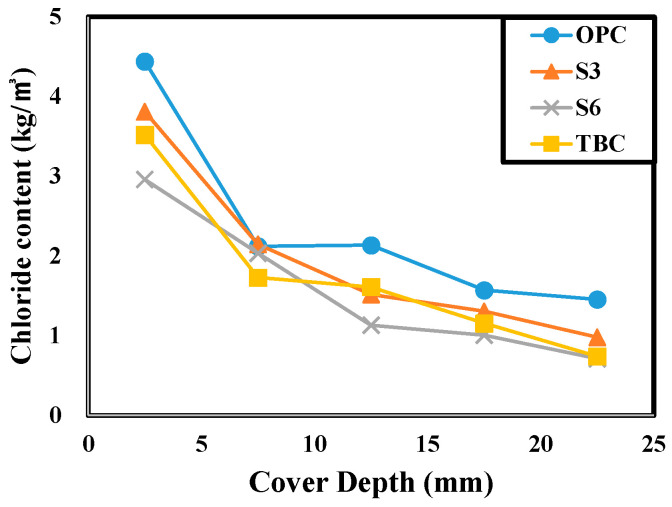
Variation of chloride content according to cover depth.

Figure 11 below shows the change in chloride content according to the cover depth.

The concrete chloride content tended to decrease as the cover depth increased at all of the experiment levels, and the chloride content in concrete decreased as the cement admixture replacement rate increased. Among the chloride content measurement results, the chloride content between the 20 mm cover depth with rebar corrosion and the 25 mm cover depth without rebar corrosion was estimated as the critical chloride content that induces corrosion of rebars embedded in concrete. From highest to lowest, the calculated critical chloride content for rebar corrosion can be ranked in the order of OPC 1.46, S3 0.98, TBC 0.74, and S6 0.71 kg/m^3^. This shows that critical chloride content for rebars embedded in concrete decreases as the replacement rate of GGBFS or FA increases. Compared to the specimen using 100% OPC, the critical chloride content of the specimen using OPC 40% + GGBFS 60% decreased by 51.37%.

This is because as the replacement rate of GGBFS or FA increases, the Ca(OH)_2_ produced by hydrated OPC stimulates a latent hydraulic reaction or is consumed by the pozzolanic reaction, so the total amount of Ca(OH)_2_ in the concrete decreases and the pH of concrete decreases accordingly. The reduced amount of Ettringite and Ca(OH)_2_ produced by the decrease in the absolute quantity of cement also has a direct effect [27,28,29,30,31,32,33,34,35]. Particularly when using FA, its effect was greater than that of using GGBFS. The critical chloride content for rebar corrosion seems to have decreased because the pH of concrete decreased, and the performance of the passive film formed on the surface of the rebars embedded in the concrete was degraded or destroyed. These results should be verified in the future through micro-analysis and electrochemical analysis.

### 4.5. Result of Service Life on RC Structure by Concrete Binder Type

This study evaluated the service life against chloride attack for reinforced concrete structures with 40 mm concrete cover using the results of assessing the concrete chloride ion diffusion coefficient according to the admixture replacement rate and admixture type measured in Section 4.2 and the critical chloride content in the Korean concrete standard specification (1.2 kg/m^3^), and the results are shown in the table below. In addition, Table 9 and Table 10 show the results of evaluating the service life applying the critical chloride content for rebar corrosion calculated in Section 4.4 of this study.

**Figure 12:**
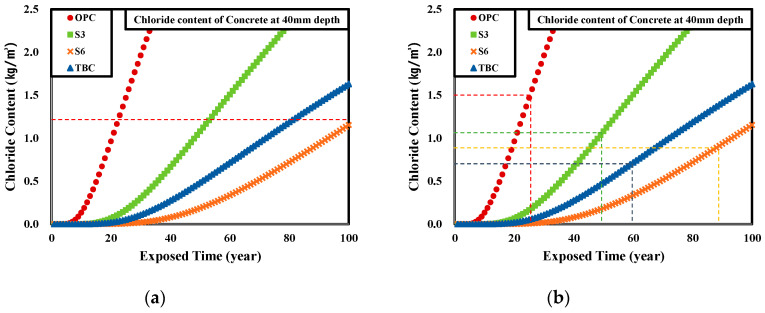
Variation of service life on the RC structure by concrete binder type: (**a**) Before (Korean standard) critical chloride content and (**b**) improved critical chloride content.

Based on the results shown above, the Figure 12 shows the durability performance of reinforced concrete structures according to the critical chloride content for rebar corrosion.

When the critical chloride content in the Korean concrete standard specification (1.2 kg/m^3^) is applied, the service life of the structures can be evaluated as 26 years for OPC concrete, 53 years for S3 concrete, 80 years for TBC concrete, and 102 years for S6 concrete. However, after critical chloride content according to the admixture replacement rate and the type of admixture is applied, the service life of the structures changes to 26 years for OPC concrete, 51 years for S3 concrete, 60 years for TBC concrete, and 89 years for S6 concrete. In the case of OPC, the service life tended to increase compared to when the conventional critical chloride content in the Korean specification was applied, but in general, the service life tended to decrease when replacing cement with admixtures.

This is because the service life evaluation includes the decrease in the absolute quantity of cement as the admixture replacement rate increases, and the decrease in the anticorrosive coating of the rebars due to the decrease in pH, so the evaluation was more accurate than in the case of applying the traditional critical chloride content for rebar corrosion.

Therefore, using the results of this study to evaluate the durability performance of reinforced concrete structures against chloride attack or to perform durability design should produce more accurate results than using standards according to the conventional concrete specifications.

## 5. Conclusions

This study evaluated the compressive strength, chloride ion diffusion coefficient, and critical chloride content of rebar corrosion according to the concrete binder type, and used the results to predict and compare the service life. The experiment level of this study consisted of four types: OPC 100%, OPC 70% + GGBFS 30%, OPC 40% + GGBFS 60%, OPC 40% + GGBFS 40% + TBC 20%, and the results are as follows.

- By evaluating the compressive strength of concrete, it was determined that the compressive strength of concrete decreased as the additive replacement ratio was increased. This tendency was more pronounced when GGBFS and FA were used together than when GGBFS was used alone. This is because the latent hydraulic reaction of GGBFS or the pozzolanic reaction of FA did not occur properly, as the amount of Ca(OH)_2_ produced decreased due to the reduced quantity of cement and the lack of curing water under air-dry curing conditions. When cement is replaced with additive in concrete, attention to curing conditions is required to increase the performance of concrete.
- By measuring the chloride ion diffusion coefficient of concrete, it was found that the chloride ion diffusion coefficient decreased as the additive replacement ratio increased. It is judged that the pore-filling effect of concrete was due to the high fineness of GGBFS and FA. This was considered to be due to the effect of blocking the penetration of chloride ions while filling the capillary pores according to the formation of C–S–H hydrate by the latent hydraulic properties.
- Through evaluating the critical chloride content of the rebars embedded in concrete, it was found that the critical chloride content to induce rebar corrosion decreased as the admixture replacement rate increased. This was because the pH of concrete was reduced before hardening as the absolute quantity of cement decreased, and the performance of the passive film formed on the surface of the rebars was reduced.
- Applying the concrete chloride ion diffusion coefficient according to the additive replacement ratio and the additive type and the critical chloride content that initiates rebar corrosion in this study will enable a more accurate evaluation and prediction of service life compared to the use of conventional standards (0.4% of binder). As a result, the calculated service life of concrete was S3 89 years, TBC 60 years, S3 51 years, and OPC 26 years.
- It was confirmed that the service life of reinforced concrete is more affected by the chloride ion diffusion coefficient than the critical chloride content. However, it was confirmed that the effect of reducing the amount of critical chloride content of reinforced concrete according to the additive replacement ratio. In order to accurately design the durability of reinforced concrete structures using additives, it is considered that continuous research on critical chloride content is necessary.

## Figures and Tables

**Table 1:** Chemical compositions of cement and additives.

Name	Chemical Compositions (%)									
	SiO_2_	Al_2_O_3_	TiO_2_	Fe_2_O_3_	CaO	MgO	SO_3_	K_2_O	Etc.	* L.O.I.
OPC	19.74	5.33	0.30	2.93	61.74	3.78	2.47	0.89	2.82	2.3
GGBFS	33.35	13.36	0.59	0.33	44.62	4.12	2.69	0.41	0.53	0.1
FA	52.66	21.44	0.92	9.20	5.01	2.01	0.27	1.13	7.29	3.86

* L.O.I.: Loss On Ignition.

**Table 2:** Concrete mix proportion according to an experimental level.

Name	Unit Weight (kg/m^3^)							Unit Weight (g/m^3^)	
	W	C	GGBFS	FA	S1 ^1^	S2 ^2^	G ^3^	S.P.	A.E.
OPC	180	300	–	–	640	273	867	3007	93
S3	180	210	90	–	640	273	867	2400	120
S6	180	120	180	–	633	267	867	2100	250
TBC	180	120	120	60	620	267	867	2100	1000

^1^ Crushed Sand, ^2^ Washed Sea Sand, ^3^ Maximum size of G: 13 mm.

**Table 3:** Test voltage and duration for concrete with normal binder content [59].

Initial Current *I*_30V_ (with 30V) (mA)	Applied Voltage *U* (after Adjustment) (V)	Possible New Initial Current *I*_0_ (mA)	Test Duration (H)
*I*_0_ < 5	60	*I*_0_ < 10	96
5 ≤ *I*_0_ < 10	60	10 ≤ *I*_0_ < 20	48
10 ≤ *I*_0_ < 15	60	20 ≤ *I*_0_ < 30	24
15 ≤ *I*_0_ < 20	50	25 ≤ *I*_0_ < 35	24
20 ≤ *I*_0_ < 30	40	25 ≤ *I*_0_ < 40	24
30 ≤ *I*_0_ < 40	35	35 ≤ *I*_0_ < 50	24
40 ≤ *I*_0_ < 60	30	40 ≤ *I*_0_ < 60	24
60 ≤ *I*_0_ < 90	25	50 ≤ *I*_0_ < 75	24
90 ≤ *I*_0_ < 120	20	60 ≤ *I*_0_ < 80	24
120 ≤ *I*_0_ < 180	15	60 ≤ *I*_0_ < 90	24
180 ≤ *I*_0_ < 360	10	60 ≤ *I*_0_ < 120	24
*I*_0_ ≥ 360	10	*I*_0_ ≥ 120	6

**Table 4:** ASTM C 876 criteria for corrosion.

Potential of Rebar (mV)				Corrosion Probablity
CSE	SCE	SHE	MnO_2_	
<−500	<−426	<−184	<−665	Severe
<−350	<−276	<−34	<−515	90% ↑
−350~−200	−276~−126	−34~+116	−515~−419	50% ↓
>−200	>−126	>+116	>−419	10% ↓

**Table 5:** Result of concrete compressive strength tests.

Name	Compressive Strength (MPa)			Slump (mm)	Air Content (%)
	3d	7d	28d		
OPC	18.0	23.2	29.2	196	3.6
S3	13.6	20.9	28.2	195	4.2
S6	11.8	20.9	27.4	200	4.5
TBC	8.4	15.4	21.8	200	3.2

**Table 6:** Result of chloride ion diffusion of concrete.

Name	Chloride Ion Diffusion Coefficient (28 days) (×10^−12^ m^2^/s)	Compressive Strength (28 days) (Mpa)
OPC	37.03	29.2
S3	15.67	28.2
S6	8.10	27.4
TBC	13.04	21.8

**Table 7:** Surface image of rebar after OCP monitoring experiment.

Name	OPC	S3	S6	TBC
Surface Image	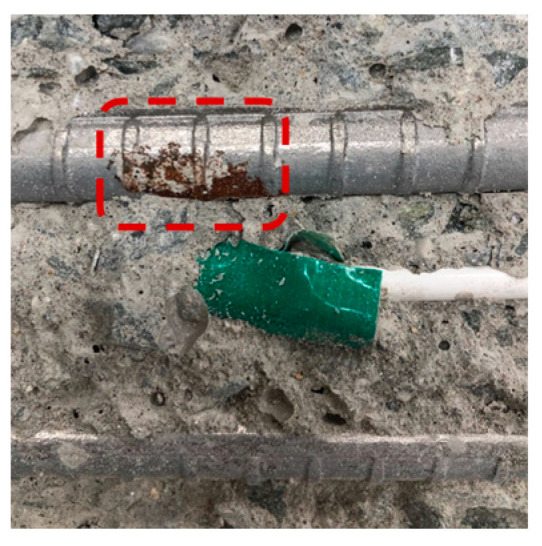	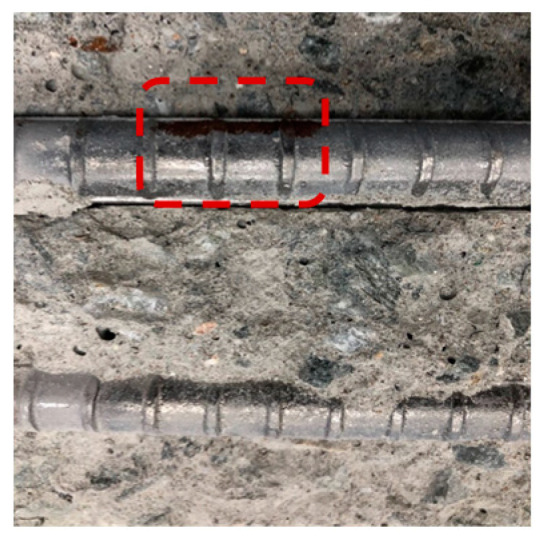	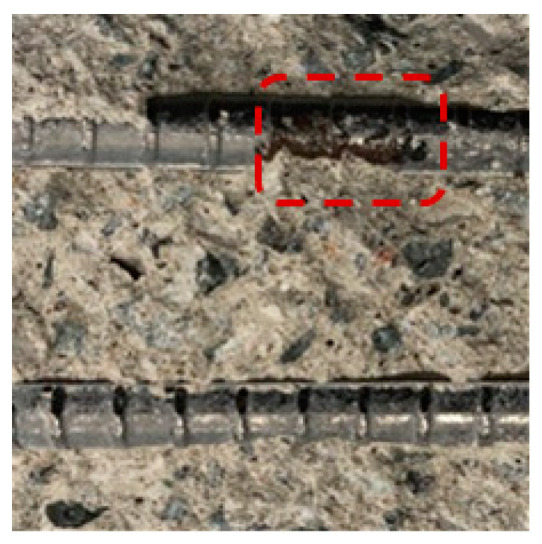	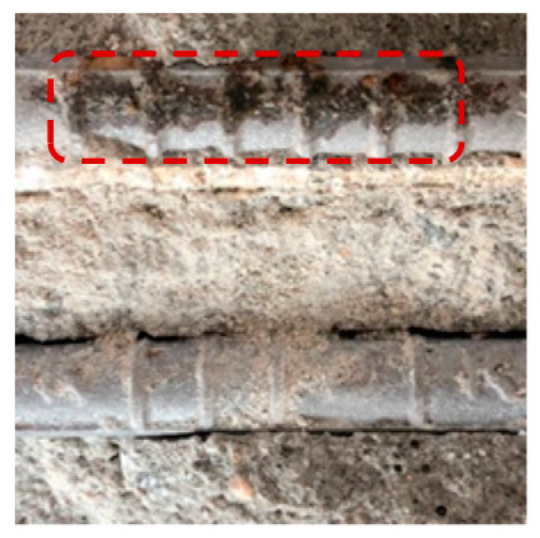

**Table 8:** Result of total chloride measurement.

Cover Depth (mm)	Amount of Chloride Content (kg/m^3^)			
	OPC	S3	S6	TBC
0–5	4.44	3.81	2.96	3.52
5–10	2.12	2.15	2.03	1.73
10–15	2.14	1.52	1.13	1.61
15–20	1.57	1.31	1.01	1.16
20–25	1.46	0.98	0.71	0.74

**Table 9:** The service life of the RC structure by binder type with Korean standard critical chloride content.

Name	Chloride Ion Diffusion Coefficient (10^−12^ m^2^/s)	Critical Chloride Content (kg/m^3^)	Service Life (Years)
OPC	37.03	1.2	23
S3	15.67	1.2	53
S6	8.10	1.2	102
TBC	13.04	1.2	80

**Table 10:** Service life of the RC structure by binder type with improved critical chloride content.

Name	Chloride Ion Diffusion Coefficient (10^−12^ m^2^/s)	Critical Chloride Content (kg/m^3^)	Service Life (Years)
OPC	37.03	1.53	26
S3	15.67	1.11	51
S6	8.10	0.91	89
TBC	13.04	0.72	60
